# Impact of HuR inhibition by the small molecule MS-444 on colorectal cancer cell tumorigenesis

**DOI:** 10.18632/oncotarget.12189

**Published:** 2016-09-22

**Authors:** Fernando F. Blanco, Ranjan Preet, Andrea Aguado, Vikalp Vishwakarma, Laura E. Stevens, Alok Vyas, Subhash Padhye, Liang Xu, Scott J. Weir, Shrikant Anant, Nicole Meisner-Kober, Jonathan R. Brody, Dan A. Dixon

**Affiliations:** ^1^ Department of Cancer Biology, University of Kansas Medical Center, Kansas City, KS, USA; ^2^ Department of Pharmacology, University of Kansas Medical Center, Kansas City, KS, USA; ^3^ Department of Surgery, University of Kansas Medical Center, Kansas City, KS, USA; ^4^ University of Kansas Cancer Center, University of Kansas Medical Center, Kansas City, KS, USA; ^5^ Department of Surgery, Sidney Kimmel Cancer Center, Thomas Jefferson University, Philadelphia, PA, USA; ^6^ Maharashtra Cosmopolitan Education Society's ISTRA, Azam Campus, University of Pune, India; ^7^ Department of Molecular Biosciences, University of Kansas, Lawrence, KS, USA; ^8^ Novartis Institutes for Biomedical Research, Basel, Switzerland

**Keywords:** HuR, MS-444, AU-rich elements, RNA stability, colon cancer

## Abstract

Colorectal cancer (CRC) is the third most common cancer and a leading cause of cancer-related mortality. Observed during CRC tumorigenesis is loss of post-transcriptional regulation of tumor-promoting genes such as COX-2, TNFα and VEGF. Overexpression of the RNA-binding protein HuR (ELAVL1) occurs during colon tumorigenesis and is abnormally present within the cytoplasm, where it post-transcriptionally regulates genes through its interaction with 3′UTR AU-rich elements (AREs). Here, we examine the therapeutic potential of targeting HuR using MS-444, a small molecule HuR inhibitor. Treatment of CRC cells with MS-444 resulted in growth inhibition and increased apoptotic gene expression, while similar treatment doses in non-transformed intestinal cells had no appreciable effects. Mechanistically, MS-444 disrupted HuR cytoplasmic trafficking and released ARE-mRNAs for localization to P-bodies, but did not affect total HuR expression levels. This resulted in MS-444-mediated inhibition of COX-2 and other ARE-mRNA expression levels. Importantly, MS-444 was well tolerated and inhibited xenograft CRC tumor growth through enhanced apoptosis and decreased angiogenesis upon intraperitoneal administration. *In vivo* treatment of MS-444 inhibited HuR cytoplasmic localization and decreased COX-2 expression in tumors. These findings provide evidence that therapeutic strategies to target HuR in CRC warrant further investigation in an effort to move this approach to the clinic.

## INTRODUCTION

The lifetime risk of developing colorectal (CRC) cancer is ~5% for both men and women in the USA [1]. Various genetic alterations have been identified that promote the initiation and progression of colon tumorigenesis. Facilitating this process is the dysregulation of gene regulatory mechanisms that can modulate cell growth and inflammation. For instance, in normal intestinal epithelium, levels of pro-oncogenic factors are controlled through 3′-UTR AU-rich elements (AREs) that target mRNAs for rapid turnover, a process known as ARE-mediated mRNA decay [2]. However, this mechanism of gene regulation is compromised during colorectal tumorigenesis, thereby allowing for selective overexpression of tumor promoting factors such as COX-2 [3–6].

A key factor that mediates ARE-mRNA stability is HuR (ELAVL1), which is a member of a family of proteins analogous to the *Drosophila* embryonic-lethal abnormal vision (ELAV) proteins [7]. HuR has been previously shown to bind to its targets via ARE motifs and promote increased mRNA stability [8–10]. In normal intestinal epithelium, HuR is localized predominantly in the nucleus (>90%) and can shuttle between the nucleus and cytoplasm [4, 8, 11, 12]. However, HuR overexpression and cytoplasmic localization is observed in 76% of colorectal adenomas and 94% of colorectal adenocarcinomas [12], allowing for aberrant stabilization of ARE-containing oncogenic mRNAs.

Previous work has identified HuR as a central protein in the process of tumorigenesis. HuR has been shown to increase cell division by enhancing the stability of several mRNAs that regulate growth and proliferation, such as cyclin A, cyclin B1 and c-fos [13, 14]. Additionally, HuR regulates key carcinogenic processes via its stabilization of the pro-inflammatory and angiogenic factors such as TNF-α, COX-2, and VEGF, among others [4, 10, 12, 15, 16]. Through its pleiotropic effects on tumor-promoting gene expression, CRC tumor-derived HuR can be viewed as a central node in promoting pathogenic gene expression necessary for the various hallmarks of cancer [7, 17].

In light of its central role in tumorigenesis and its differential expression between normal and cancerous tissues, HuR has emerged as an attractive therapeutic target for cancer [18–20]. Efforts to identify novel HuR inhibitors have led to the discovery of small molecule compounds derived from microbial and plant extracts, among them MS-444 [21]. Interestingly, MS-444 was shown to interfere with HuR binding to its target mRNAs and influence HuR cytoplasmic localization. Such properties resulted in growth inhibition and loss of cytokine expression in inflammatory cell models [21].

In this report, we demonstrate that cytoplasmic HuR localization sensitizes colon cancer cells to the growth-inhibitory effects of MS-444, while non-transformed intestinal epithelial cells are refractory to these effects. Additionally, we demonstrate that HuR inhibition by MS-444 results in inhibition of COX-2 expression both *in vitro* and *in vivo*. Importantly, MS-444 efficiently inhibited CRC tumor growth in pre-clinical models, thereby underscoring HuR inhibition as a viable and cancer cell-specific therapeutic strategy.

## RESULTS

### MS-444 inhibits the growth of CRC cells

**Figure 1:**
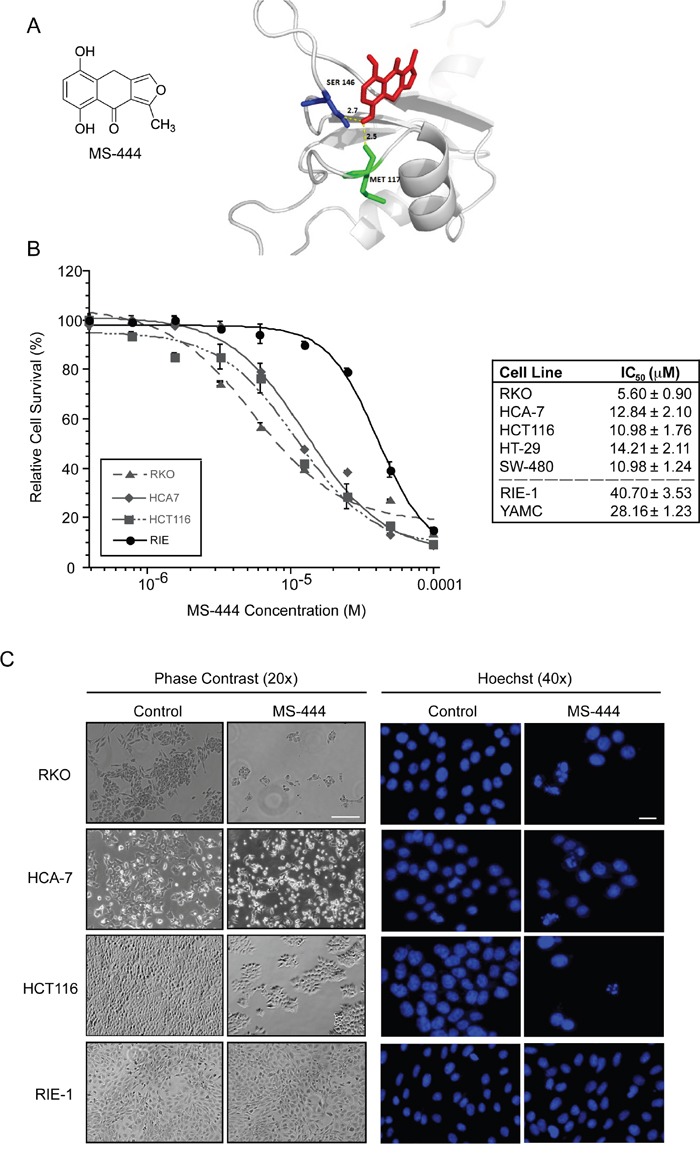
MS-444 inhibits colorectal cancer cell proliferation **A.** MS-444 structure and molecular docking with HuR RRM1/2 (PDB ID: 4EGL). **B.** Human colorectal cancer cell lines RKO, HCA-7, and HCT116, HT-29, and SW480 and the non-transformed intestinal epithelial cell lines RIE-1 and YAMC were treated with varying concentrations of MS-444 for 48 hr. Cell survival was measured by MTT assay after incubation of cells for 48 hr with MS-444. Relative cell survival was calculated as percentage normalized to DMSO vehicle-treated cells and plotted to determine IC_50_. Data is represented as average of 4 independent experiments ± SEM. **C.** RKO, HCA-7, HCT116 and RIE-1 cells were treated with 10 μM MS-444 or vehicle control for 48 hr and subjected to phase contrast microscopy and 1 μg/ml Hoechst 33342 staining to visualize cellular and nuclear morphology, respectively. Bars = 10 μm.

A high-throughput based biochemical screen had previously identified the chrysanthone-like compound MS-444 (Figure 1A) as a specific inhibitor of HuR binding of ARE-RNAs [21]. MS-444 is produced by gram-positive bacteria *Micromonospora* and originally characterized as myosin light chain (MLC) kinase inhibitor [22, 23]. Molecular docking between the conserved tandem RNA recognition motif (RRM) domains 1 and 2 that are responsible for ARE-binding activity [24–28] and MS-444 predicted a binding energy of −4.6 kcal/mol with hydrogen bonding within the RRM2 domain at residues Ser^146^ and Met^117^ with bond lengths of 2.7 Å and 2.5 Å, respectively (Figure 1A). This possible binding mode is consistent with previous data showing that MS-444 inhibits an RRM1-2 truncation mutant of HuR [21].

To determine the effects of inhibiting HuR by MS-444, CRC cells that display HuR overexpression (HCT116, HCA-7, RKO, HT-29, and SW480) [3, 4, 12, 29, 30] were treated with various concentrations (1–100 μM) of MS-444 for 48 hr. Growth inhibition was observed in all CRC lines with IC_50_ values ranging from 5.60 μM to 14.21 μM with observable effects seen at 10 μM MS-444 (Figure 1B-1C). Contrasting effects were observed using non-transformed small intestinal (RIE-1) and colonic (YAMC) epithelial cells [31]. Both cell types display properties of normal intestinal epithelial cells (e.g. polarized growth, formation of tight adherens junctions, and contact-mediated growth inhibition) and are proficient in ARE-mRNA decay [32–34]. Both non-transformed cell lines were ~3- to 4-fold less responsive to MS-444-mediated growth inhibition, with IC_50_ values of 40.70 μM and 28.16 μM (*P <* 0.05) (Figure 1B). Additionally, examination of nuclear morphology by Hoechst staining showed MS-444-treated CRC cells displayed apoptotic features, such as fragmented and condensed chromatin, while RIE-1 nuclei remained intact in the presence of MS-444 (Figure 1C).

**Figure 2:**
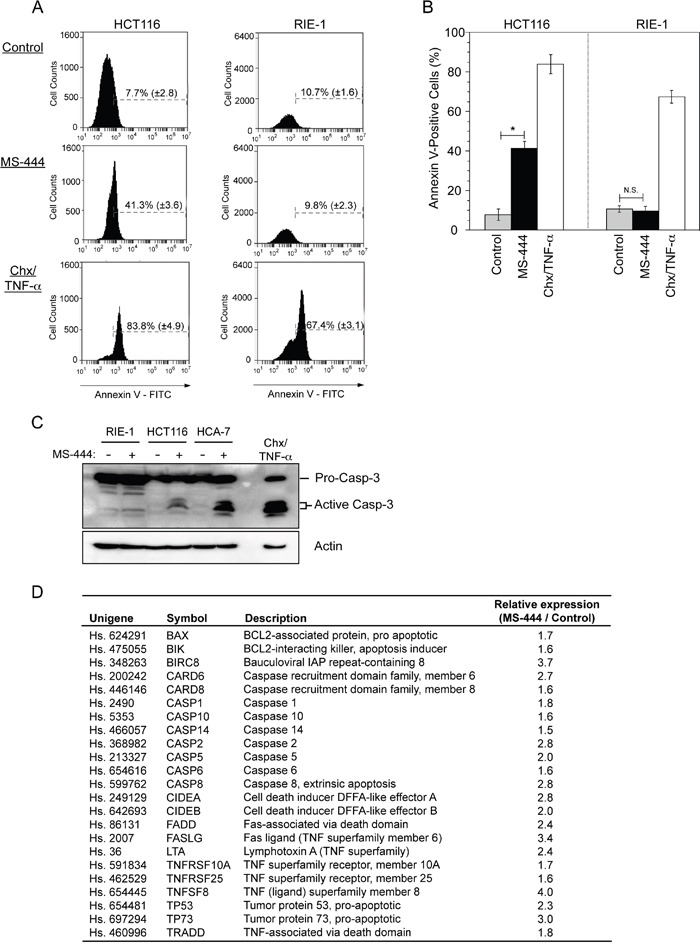
MS-444 selectively promotes apoptosis in CRC cells **A-B.** HCT116 and RIE-1 cells were treated with 10 μM MS-444 for 48 hr and stained Annexin V-FITC and PI to detect apoptotic cell death. Cells treated with 5 μg/ml cycloheximide and 10 ng/ml TNF-α for 8 hr were used as positive controls. Flow cytometry analysis was done to determine the percentage of Annexin V-positive cells. Bar graphs representing the observed percentages of Annexin-V-positive cells with percentages shown as averages of 3 experiments ± SEM. **C.** RIE-1, HCT116, and HCA-7 cells were treated with 10 μM MS-444 for 48 hr and detected for cleaved caspase 3 by western blot. Actin was used as a loading control. **D.** SW480 cells treated with 10 μM MS-444 for 48 hr were subjected to a qPCR apoptosis array. Genes induced >1.5-fold are shown as fold change relative to control-treated cells and are the average of 3 experiments.

Previous studies have demonstrated HuR overexpression to be anti-apoptotic through its influence on the expression of multiple pro-survival mRNAs [35, 36]. To determine if HuR inhibition by MS-444 promoted apoptosis, HCT116 colon cancer cells and non-transformed RIE-1 cells were treated with 10 μM MS-444 for 48 hr and assayed by flow cytometry for annexin V detection using propidium iodide to identify and exclude late apoptotic and necrotic cells. Treatment of cells with cycloheximide and TNF-α served as a positive control. While MS-444 treatment triggered a strong (>5-fold) apoptotic response in HCT116 cells, RIE-1 cells were refractory to this effect at identical MS-444 concentrations (Figure 2A-2B). Similar to these results, MS-444 treatment promoted caspase 3 activation in CRC cells HCT116 and HCA-7 with no detectable levels of caspase 3 cleavage observed in RIE-1 cells (Figure 2C). A significant increase in pro-apoptotic gene expression was observed in MS-444-treated SW480 and HCT116 CRC cells (Figure 2D and data not shown, respectively). Previous studies have demonstrated that siRNA-mediated knockdown of HuR can promote apoptosis [35, 36]. Consistent with this, siRNA knockdown of HuR in HCT116 cells resulted in apoptosis. However, apoptosis induction by HuR knockdown was less robust (15.5%) when compared to 10 μM MS-444 treatment (41.3%) (Supplementary Figure S1A-S1B, Figure 1A-1B). Together, these results demonstrate the ability of MS-444 to promote apoptosis selectively in CRC cancer cells and not in non-transformed intestinal epithelial cells within the low micromolar concentration range, thus providing a potential therapeutic window.

### Cytoplasmic localization of HuR sensitizes cells to MS-444

**Figure 3:**
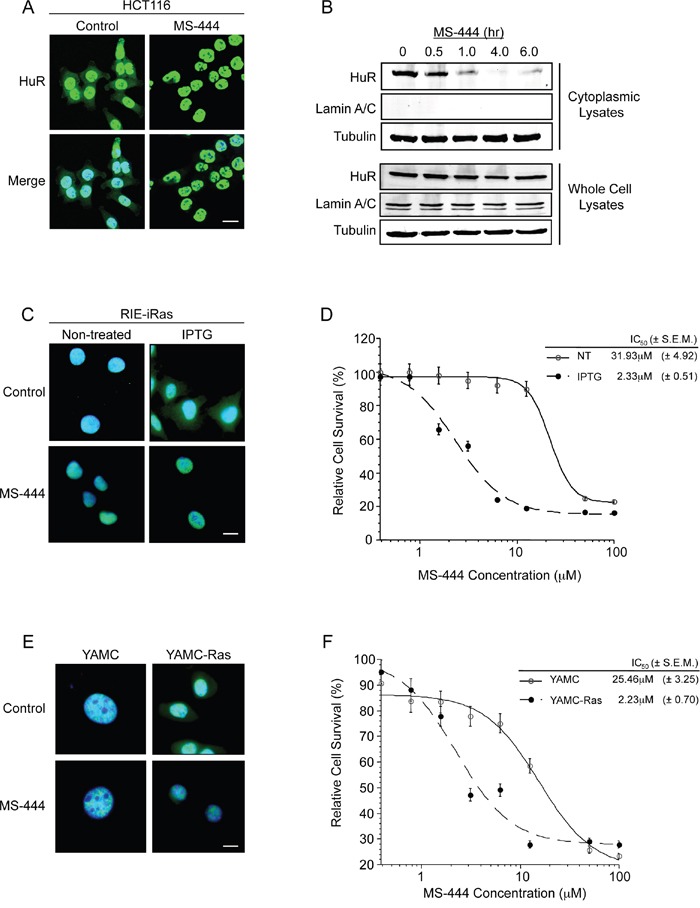
MS-444 inhibits HuR cytoplasmic localization **A.** HCT116 cells treated with 10μM MS-444 for 6 hr were subjected to HuR immunofluorescence analysis (shown in green). DAPI (shown in blue) was used to visualize nuclei. **B.** HCT116 cells were treated with 10μM MS-444 for the indicated times. Cytoplasmic and whole cell lysates were probed for HuR, along with cytoplasmic α-tubulin and nuclear Lamin A/C markers. **C.** RIE-iRas cells were untreated or with 5 mM IPTG for 24 hr to induce oncogenic Ras expression, followed by 10 μM MS-444 for 8 hr. HuR was detected by immunofluorescence (green) along with DAPI was used to visualize nuclei (merged images shown). **D.** RIE-iRas were grown in the presence or absence of 5 mM IPTG for 24 hr and then treated with indicated amounts of MS-444 for 48 hr. Relative cell survival was performed by MTT assay and is represented as average of 4 independent experiments ± SEM. **E.** YAMC and YAMC-Ras cells were grown under non-permissive conditions at 37°C and treated with 10 μM MS-444 for 8 hours. HuR localization was assayed by immunofluorescence. **F.** MTT assay of YAMC and YAMC-Ras treated with MS-444 for 48 hr under non-permissive conditions. Bars = 10 μm.

Rapid ARE-mRNA decay occurs primarily in the cytoplasm, however the presence of cytoplasmic HuR in CRC cells impedes this process [3, 4, 6, 12, 37, 38]. To test the effects of MS-444 on HuR behavior in CRC cells, immunofluorescence and western blot analyses of HuR expression revealed that 10 μM MS-444 induced nuclear HuR localization in HCT116 cells with loss of cytoplasmic HuR occurring after 1 hr of treatment (Figure 3A-3B). This rapid change in localization was not due to HuR loss since treatment with 10 and 50 μM MS-444 up to 24 hr showed no differences in level of HuR expression in whole cell lysates (Figure 3B, Supplementary Figure S3A) and HuR cleavage products were not observed (data not shown). The effects of MS-444 also were specific to HuR since subcellular localization of endogenous (p38 MAPK, Erk1/2, and Akt) proteins were not impacted (Supplementary Figure S2). Furthermore, MS-444 was not observed to impact myosin light chain (MLC) kinase levels [22, 23] or MAPK signaling up to 100 μM (Supplementary Figure S3B-S3C).

We have previously demonstrated the ability of oncogenic Ras to influence HuR-mediated post-transcriptional regulation of COX-2 and ARE-containing mRNAs during intestinal cell transformation [32–33]. In this context, we sought to assess the specificity of MS-444 on oncogenic Ras-transformed cells and its effect on HuR localization. We examined the effects of MS-444 on non-transformed cells and isogenic variants (RIE-iRAS) that are transformed by oncogenic Ras. RIE-iRas cells are RIE-1 containing an IPTG-inducible cDNA of Ras^V12^, and when cultured in the presence of IPTG for 24 hr to induce oncogenic Ras, undergo epithelial-to-mesenchymal transition (EMT) [33, 34]. Shown in Figure 3C, immunofluorescence analysis of RIE-iRas cells expressing oncogenic Ras showed cytoplasmic localization of HuR, whereas in non-transformed cells HuR is predominantly nuclear. RIE-iRas cells were then cultured in the presence and absence of IPTG for 24 hr, after which 10 μM MS-444 was added. After 8 hr of MS-444 treatment, HuR localization to the nucleus was detected (Figure 3C). To determine if Ras-transformed cells exhibited a higher sensitivity to MS-444, growth assays were performed. RIE-iRas expressing oncogenic Ras exhibited >13-fold increased sensitivity to MS-444 with an IC_50_ of 2.33 μM, as compared to 31.93 μM in non-transformed cells (Figure 3D). Similar results were obtained using conditionally immortalized YAMC colonocytes and Ras-transformed YAMC cells expressing the v-Ha-Ras oncogene [39] (YAMC-Ras, Figure 3E-3F), further indicating that MS-444 sensitivity correlates with cytoplasmic HuR.

### MS-444 regulates COX-2 expression in CRC cells

**Figure 4:**
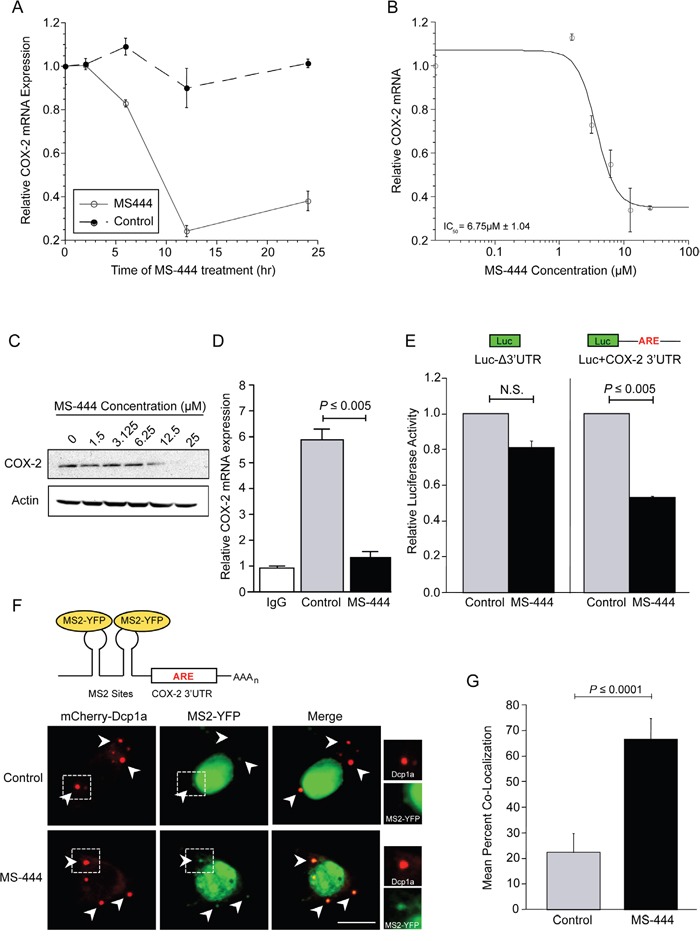
MS-444 inhibits COX-2 expression and promotes ARE-containing mRNA trafficking to P-bodies **A.** HCA-7 cells were treated with 12 μM MS-444 or a vehicle control for the indicated times. COX-2 mRNA levels were assayed by qPCR using GAPDH as a loading control and normalized to non-treated cells. Each value represents an average of triplicates ± SEM. **B.** HCA-7 cells were with the indicated concentrations of MS-444 for 8 hr and COX-2 mRNA levels were assayed by qPCR. The data was fitted to a dose-response curve to determine the IC_50_ with respect to COX-2 mRNA expression. Each data point represents an average of triplicates ± SEM. **C.** HCA-7 cells were treated with the indicated concentrations of MS-444 for 8 hr and COX-2 protein expression was assayed by western blot. Actin was used as a loading control. **D.** HCA-7 cells were treated with DMSO (control) or 10 μM MS-444 for 6 hr and RNP-IP of HuR or control IgG was done to isolate mRNAs bound by HuR. qPCR was used to quantitate COX-2 mRNA levels normalized to IgG control as the average from 3 independent experiments ± SEM. **E.** HCT116 cells were transfected with luciferase reporter constructs without the COX-2 3′UTR (LucΔ3′UTR) or fused to the full-length COX-2 3′UTR and treated with 10 μM MS-444 for 8 hr. Luciferase activity was normalized to total protein activity and is the average of 3 experiments ± SEM. N.S., not significant. **F.** Representation of the MS2 system showing the reporter mRNA containing MS2 sites present upstream of the ARE-containing COX-2 3′UTR bound by YFP-tagged MS2 binding protein, allowing for fluorescent visualization of the mRNA. HCT116 cells were transfected with the MS2 dual plasmid system using the MS2-COX-2 3′UTR mRNA expression constructs, along with MS2-YFP and mCherry-tagged Dcp1a to visualize mRNA and P-bodies, respectively. Cells were treated with 10 μM MS-444 for 8 hr and visualized for P-bodies (red) and MS2-YFP-bound mRNA (pseudo-colored green). Arrowheads indicate representative P-body signal with boxed areas enlarged on right showing Dcp1a and YFP-MS2 signal. Scale bar = 10 μm. **G.** Co-localization between the reporter MS2-COX-2 3′UTR and P-bodies is shown as percentage of P-bodies per cell exhibiting co-localization between the MS-2-bound YFP mRNA reporter and Dcp1a signal ± SEM (n = 10 cells per group).

Our prior work has shown HuR to be a key contributor to COX-2 overexpression in CRC through ARE-mediated mRNA stabilization [3, 6, 12]. To test whether HuR inhibition by MS-444 affected endogenous COX-2 levels, HCA-7 cells were treated with 12 μM MS-444 over a 24 hr time-course that revealed a loss of COX-2 mRNA occurring within 6-12 hr of treatment (Figure 4A). HCA-7 cells were then treated with increasing concentrations of MS-444 for 8 hr, and COX-2 mRNA and protein expression was assayed. COX-2 mRNA expression was significantly inhibited in a dose-dependent manner with an IC_50_ of 6.75 μM (Figure 4B), along with a similar dose-dependent attenuation in COX-2 protein observed (Figure 4C).

To establish whether the effect of MS-444 on COX-2 expression occurred through disruption of the HuR/COX-2 mRNA association, ribonucleoprotein immunoprecipitation (RNP-IP) was performed in HCA-7 cells treated with 10 μM MS-444 for 6 hr and immunoprecipitation of cytoplasmic lysates was done using an antibody against HuR. The association of COX-2 mRNA with HuR was assayed by qPCR of COX-2 mRNA in immunoprecipitates. As shown in Figure 4D, COX-2 mRNA was significantly enriched in the HuR IP samples in vehicle treated cells compared to RNP-IP samples using control IgG. In contrast, a 4-fold reduction in COX-2 mRNA was observed in HuR immunoprecipitates from MS-444 treated cells. To determine if the effects on COX-2 occurred via the ARE-containing COX-2 3′UTR [10], HCT116 cells were transfected with luciferase reporter containing the COX-2 3′UTR and treated with 10 μM MS-444 for 8 hr (Figure 4E), MS-444 promoted approximately 2-fold inhibition in luciferase expression in the presence of the ARE-containing 3′UTR and did not significantly influence expression of a control reporter (LucΔ3′UTR). Consistent with these observations, MS-444 did not impact levels of non-ARE-containing mRNAs COX-1 and β-actin (Supplementary Figure S4A), further supporting that MS-444 regulates COX-2 expression via the 3′UTR.

In non-transformed intestinal epithelial cells, COX-2 and ARE-mRNAs are degraded in discrete cytoplasmic RNA granules known as processing bodies (P-bodies) [31]. To determine the effects of MS-444 upon P-body formation and mRNA localization to P-bodies, we utilized the MS2 dual plasmid system for fluorescent RNA visualization [31, 40]. HCT116 cells were transfected with the reporter construct pMS2-COX-2 3′UTR, which expressed a chimeric RNA consisting of 12 tandem MS2 RNA hairpins and the COX-2 3′UTR (Figure 4F). Cells were co-transfected with pMS2-YFP, which expresses YFP-tagged MS2-binding protein that contains a nuclear localization signal (NLS), allowing for visualization of the chimeric MS2-COX-2 3′UTR RNA (as the complex MS2-YFP/MS2-COX-2 3′UTR). P-bodies were visualized by co-transfecting the mCherry-tagged P-body marker Dcp1a [31]. As shown in Figure 4F (right panels), treatment with MS-444 for 8 hr did not impact P-body numbers or size, with approximately 6 P-bodies per cell detected in both control and MS-444-treated cells (Supplementary Figure S4B). Due to the presence of the NLS in the MS2-YFP protein, the majority of MS2-YFP signal appeared nuclear in both conditions (Figure 4F). In control-treated cells, MS2-COX-2 3′UTR RNA observed in the cytoplasm showed limited trafficking to P-bodies with co-localization occurring only in 23% of P-bodies. By contrast, treatment with MS-444 enhanced co-localization of MS2-COX-2 3′UTR RNA to Dcp1a signal, with approximately 70% of the total number of P-bodies per cell showing co-localization with the COX-2 3′UTR reporter (Figure 4F-4G). Control experiments using MS2 RNA lacking a 3′UTR (MS2-Δ3′UTR) showed no reporter RNA co-localization with Dcp1a in the presence or absence of MS-444 (data not shown). These findings indicate that MS-444 promotes COX-2 mRNA decay by allowing mRNA trafficking to P-bodies.

### MS-444 inhibition of CRC tumor growth *in vivo*

Prior examination of MS-444 acute toxicity in rats showed no lethality at concentrations of 100 mg/kg by intraperitoneal (IP) administration [41]. To determine a tolerable dose range for mice, a dose escalation study over 24 hr period was performed by IP, oral (PO), and intravenous (IV) administration. For IP and PO administration, no signs of acute toxicity were observed up to 100 mg/kg. IV administration was well tolerated up to 25 mg/kg, with some signs of toxicity (hunched posture and tremors) observed at 50 mg/kg.

**Figure 5:**
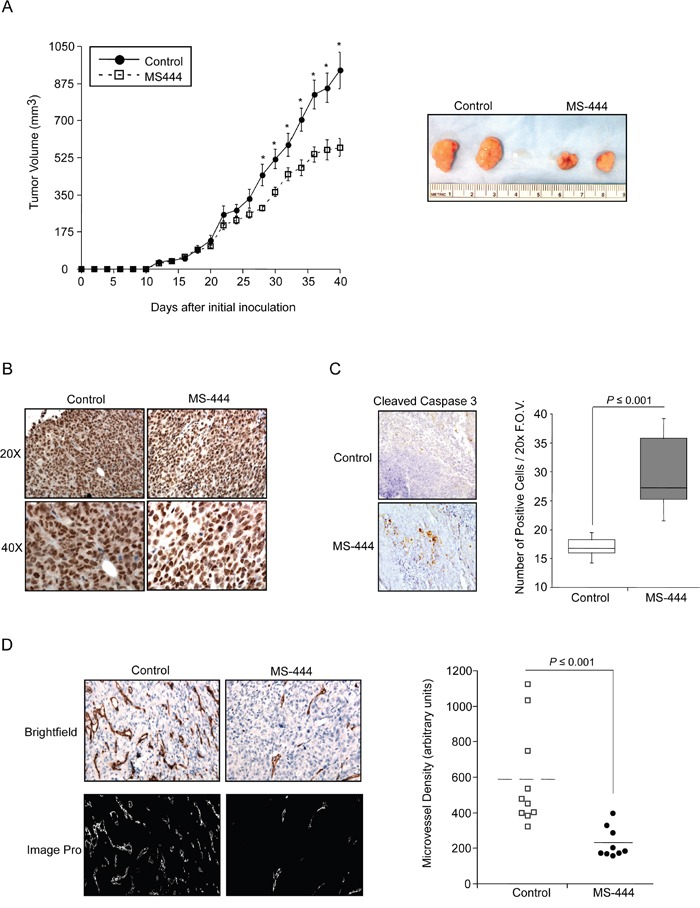
Inhibition of *in vivo* tumor growth by MS-444 **A.** Tumor growth of HCT116 cell implants in nude mice treated with 25 mg/kg MS-444 or vehicle control every 48 hr. Representative tumors excised at day 40 and are shown. *, *P* ≤ 0.05. **B.** IHC detection of HuR in MS-444-treated HCT116 xenograft tumors. Representative tissue sections were examined for HuR expression and counterstained with hematoxylin. Images were acquired at 20x and 40x magnification. **C.** IHC detection of cleaved caspase 3. Box plots show the apoptotic index determined by counting the number of cleaved caspase 3-positive cells per 20x field of view (F.O.V). **D.** HCT116 xenograft tumors were analyzed for microvessel density using anti-CD31 antibody to mark blood vessels (upper panels). To facilitate microvessel visualization and quantification, the positive CD31 signal was transposed onto a mask and depicted in white using ImagePro (lower panels). For each tumor, 4 random images were captured at 40X magnification. Each dot represents an average of all scored fields using 9-10 tumors.

To test the effects of MS-444 on CRC cell growth *in vivo*, mice bearing HCT116 cell xenografts received IP injections of MS-444 (25 mg/kg bw) or vehicle every 48 hr. Over the experiment course, mice did not display any adverse effects and maintained similar weights (Supplementary Figure S5A). Anti-tumor effects of MS-444 were observed with approximately 1.7-fold reduction in tumor size (Figure 5A). The effects of MS-444 to inhibit cytoplasmic HuR localization were also observed in treated tumors (Figure 5B). To determine if MS-444 treatment attenuated tumor growth through enhanced apoptosis, sections were stained for caspase 3 cleavage. Shown in Figure 5C, MS-444 treated tumors exhibited a higher amount of cleaved caspase 3-positive cells with significantly heightened apoptotic index. Based on cytoplasmic HuR localization supporting enhanced angiogenic gene expression and tumor angiogenesis [42, 43], we evaluated the impact of MS-444 on tumor microvessel density (MVD). HCT116 tumor sections were immunostained with anti-CD31 antibody to visualize blood vessels and MVD was determined by counting the number of blood vessels per field imaged. Mice treated with MS-444 showed a marked 2- to 3-fold decrease in MVD (Figure 5D), indicating the anti-angiogenic potential of MS-444.

### MS-444 attenuates tumor COX-2 expression

**Figure 6:**
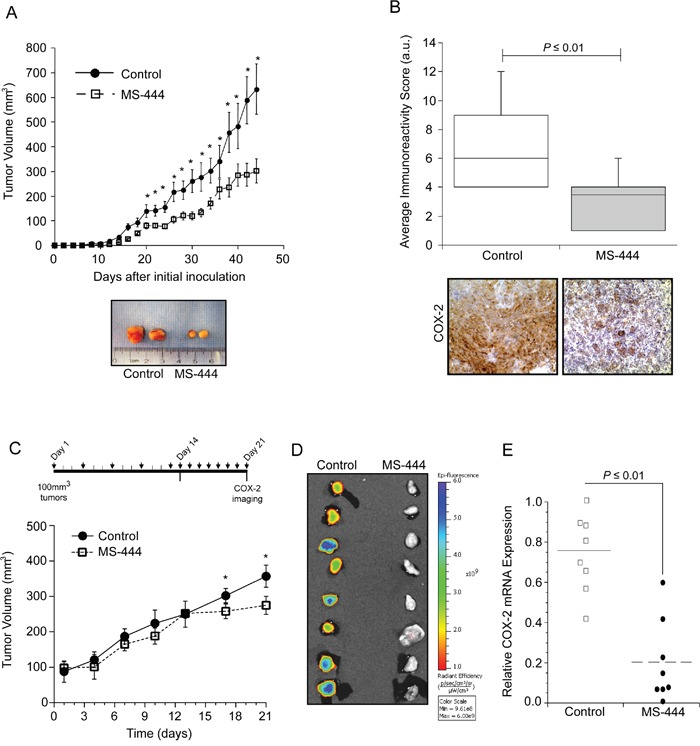
MS-444 inhibits COX-2 expression *in vivo* **A.** Tumor growth of HCA-7 cell implants in nude mice treated with 25 mg/kg MS-444 or vehicle control every 48 hr. Representative tumors excised at day 44 and are shown. *, *P* ≤ 0.05. **B.** IHC detection of COX-2. Graph indicates the average immunoreactivity scores (IRS) from stained tumor sections (n=10 tumors). **C.** Tumor growth of established HCA-7 tumors in mice treated with 25 mg/kg bw MS-444 or vehicle control by IP for 3 weeks as indicated. *, *P* ≤ 0.01. **D.** After 21 days, mice were injected with 2 mg/kg Fluorocoxib, tumors were excised and imaged to detect COX-2 expression *ex vivo*. Epi-fluorescence scale is shown. **E.** COX-2 mRNA expression of excised tumors was measured by qPCR using GAPDH as a loading control.

Based on results shown above, the ability of MS-444 to alter COX-2 expression *in vivo* was determined using HCA-7 cell xenografts that display elevated COX-2 levels and COX-2 dependent tumor growth [12, 44, 45]. Using dosing as described with HCT116 tumors, IP administration of MS-444 was more effective at inhibiting HCA-7 tumor growth without any observable adverse effects (Figure 6A, Supplementary Figure S5B). Consistent with the ability of MS-444 to inhibit COX-2 expression in HCA-7 cells (Figure 4), IHC analysis of COX-2 protein levels in HCA-7 xenograft tumors was attenuated by MS-444 (Figure 6B).

Our *in vivo* observations indicate that MS-444 impacts the growth of established tumors >100 mm^3^ (Figure 5A, Figure 6A). To further characterize this and evaluate alternative MS-444 dosing schedules, HCA-7 cell xenografts were allowed to form palpable tumors (approximately 100 mm^3^) and then treated intraperitoneally with MS-444 (25 mg/kg bw) every 72 hr for 15 days followed by a 7-day period during which they received daily injections of the same dose (Figure 6C). Under this dosing schedule, no significant change in tumor volume was observed until daily dosing at day 14 (Figure 6C). Assessment of COX-2 expression was determined by IP injection of the fluorescently-labeled COX-2 probe (Fluorocoxib) [46] on day 21. *Ex vivo* Image analysis of the freshly excised tumors revealed a significant decrease in COX-2 protein expression (Figure 6D). COX-2 mRNA analysis determined by qPCR corroborated these findings (Figure 6E), indicating that selective small molecule-based inhibition of HuR functionally impacts tumor COX-2 levels and alters tumor growth potential.

## DISCUSSION

Although somatic mutations have been closely linked to the progression model of CRC tumorigenesis [47]. CRC tumors have a disproportionate level of tumor promoting genes expressed that contain AREs within their 3′UTRs [33], and many of these mRNA transcripts have been shown to be bound and regulated by the RNA binding protein HuR [19]. Previous work in our lab demonstrated that cytoplasmic HuR localization is observed in 76% of colorectal adenomas and 94% of colorectal adenocarcinomas [12]. Moreover, increased HuR expression and cytoplasmic localization in tumors correlate with poor disease prognosis and advanced tumor staging [12, 48–51]. Thus, there are many data points from multiple laboratories over various tumor types that HuR is a strong candidate target in cancer [7, 18, 19, 52]. In this study, MS444 is used here as a tool compound for proof-of-principle for pharmacological HuR inhibition, and that targeting HuR with MS-444 has anti-tumor activity as a monotherapy in multiple pre-clinical models for CRC.

Logically, the therapeutic strategies to inhibit HuR, especially in CRC, would be to: 1) silence expression levels [53], 2) disrupt the ARE-binding activity which is mediated through the conserved RNA recognition motifs (RRM) domains of HuR, and 3) inhibit HuR nuclear to cytoplasmic translocation. In this study, we explored MS-444, originally identified as a HuR inhibitor in a confocal high-throughput screen of microbial, mycological, and plant extracts [21], wherein MS-444 was successful in disrupting binding between HuR and its ARE-containing mRNA targets. Structurally, HuR consists of two-tandem RRM domains, followed by a hinge region and a third RRM domain. The ARE-binding activity of HuR is mediated through the conserved RRM1/2 domains [24–27]. RRM1/2 may also provide an interface for HuR homodimerization, with each HuR monomer contacting a single ARE [21, 54]. Thus mechanistically, it was established that MS-444 binds HuR within its first two tandem RRM motifs and prevents HuR homodimerization, preventing HuR from trafficking to the cytoplasm, where it is known to exert its mRNA-stabilizing function [21].

Based on HuR's ability to support an anti-apoptotic network through post-transcriptional mechanisms [7, 35], we performed a focused qPCR array of apoptosis-related genes in MS-444-treated CRC cells. Expression profiling revealed an increased expression of many pro-apoptotic markers as a result of MS-444 treatment. Our data demonstrate that the expression of Fas ligand, a TNF superfamily member, is significantly increased, accompanied by elevated expression of caspase 8 and cell death effectors such as Bax and Bid. Additionally, the TNF receptor superfamily member 10A (TNFSF10A, Death receptor 4) was significantly upregulated, suggesting MS-444 triggers an apoptotic response mediated by TNF superfamily members and death receptor 4 (Figure 2D). Importantly, DR4 has recently been validated as a HuR target in another tumor system [55]. These results, along with previous findings in the field, suggest that inhibition of HuR (via MS-444 treatment) triggers a Fas-dependent signaling cascade mediated through cellular caspases, thereby resulting in cell death. An important remaining question is whether MS-444′s pro-apoptotic effect is directly due to the disruption of HuR target gene regulation. For example, previous studies have demonstrated that HuR mediates an anti-apoptotic response by repressing the expression of death receptor 5 (DR5), and now more recently DR4 [36, 55]. Consistent with these findings, siRNA-mediated knockdown and MS-444 inhibition of HuR resulted in elevated DR5/DR4 expression levels, thus promoting apoptosis (Figure 2, Supplementary Figure S1) [35, 36, 55], presumably by making the receptor more available for targeting and signaling. Interestingly, apoptosis induction by HuR knockdown was less robust when compared to MS-444 treatment. Prior work has demonstrated that during caspase-mediated apoptosis, HuR is cleaved to generate two cleavage products that switch its function from being a pro-survival factor to a promoter of apoptosis in response to lethal stress [56–58]. Of note, we did not detect the induction of HuR caspase-dependent cleavage when cells are treated with MS-444 (unpublished data D.A.D and J.R.B), indicating that MS-444 selectively targets HuR and does not influence other pathways known to promote HuR cleavage.

Studies have demonstrated that inhibition of HuR can enhance therapeutic efficacy of certain therapeutic strategies by inhibiting stressors (e.g., hypoxia, glucose deprivation, chemotherapies) that induce HuR cytoplasmic localization in pancreatic cancer cells [18, 59–61]. Our findings showing the ability of MS-444 to attenuate CRC tumor growth *in vivo* through enhanced apoptosis and decreased angiogenesis, implicate that selective targeting of these pathways in combination with MS-444 may improve the limited inhibition on tumor growth by MS-444 as a monotherapy. Further efforts to evaluate these findings in CRC models as a means to identify clinically available targeted and cytotoxic therapies that would synergize with HuR targeted therapy are in progress. These efforts, along with other modes of HuR inhibition (i.e., targeted siRNA delivery [53]), being evaluated in pre-clinical models for: 1) targeting specificity, 2) toxicity profiles, and 3) therapeutic effects against malignant phenotypes, will further support the notion that HuR is a valuable target in CRC and that pharmacological HuR inhibitors such as MS-444 warrant further investigation.

## MATERIALS AND METHODS

### Cell culture, reagents and molecular docking

Human colorectal cancer cells SW480, HT-29, HCT116, and RKO were obtained from the American Type Culture Collection (ATCC); HCA7 cells were provided by S. Kirkland (Imperial College, London, United Kingdom). Cells were maintained in Dulbecco's modified Eagle medium (DMEM) supplemented with 10% fetal bovine serum (FBS) (Hyclone). Intestinal epithelial cells lines RIE-1, RIE-iRas, YAMC, and YAMC-Ras were provided by R. D. Beauchamp (Vanderbilt University Medical Center, Nashville, TN). RIE-iRas cells were cultured as described [34] using 5 mM isopropyl β-D-1-thiogalactopyranoside (IPTG) to induce the expression of oncogenic Ras^V12^ cDNA. Conditionally immortalized colonocytes YAMC and YAMC-Ras (YAMC cells overexpressing the v-Ha-Ras oncogene) cells were maintained under permissive conditions as described [39] at 33°C in RPMI-1640 media containing 25 mM HEPES and 2 mM L-glutamine (Invitrogen) and supplemented with 5% FBS, insulin, transferrin, and selenium (ITS) (Sigma-Aldrich), and 5 U/ml of murine IFN-γ (Roche). Experiments were performed with cells at non-permissive conditions at 37°C without IFN-γ in the growth medium. MS-444 was obtained from Novartis Institutes for Biomedical Research (Basel, Switzerland) and solubilized in DMSO using an extinction coefficient of 2,200 M^−1^cm^−1^ at 328 nm.

Molecular docking was performed using the X-ray crystal structure of two methylated tandem RRM domains (RRM1/2) of HuR in their RNA-free form (PDB ID: 4EGL) [28]. MS-444 ligand was energy minimized and partial charges were added using PRODRG algorithms [62] and docked using Auto-Dock Vina software [63]. The Auto-Dock Tools graphical user interface [64] was used to add polar hydrogens and partial charges to 4EGL using Kollman United charges. Atomic solvation parameters and fragment volumes were assigned using the ADDSOL subroutine. The grid map was calculated using the auxiliary program Autogrid3. Grid maps of 120×120×120 points centered on the active site of the ligand with 0.375 A° spacing were calculated for each atom types found on the adducts. Lamarckian Genetic Algorithm (LGA) was selected for ligand conformational search. The Genetic Algorithm (GA) population size was set to 150, the maximum number of GA energy evaluations as 2500000, GA mutation rate as 0.02, GA crossover rate as 0.8 and GA docking runs was set as 100. The resulting docking conformations were clustered into families of similar conformation, with root mean square deviation (RMSD) clustering tolerance as 1.0 A°. As a rule, the lowest docking energy conformations were included in the largest cluster. Flexible torsion in the ligands was assigned with AUTOTORS, an auxiliary module for Auto-Dock Tools. The ligand was docked to obtain the best binding conformation.

### MS-444 sensitivity assay

Cells were seeded at subconfluent levels (<50% confluence) in 96-well tissue culture plates and treated with increasing concentrations of MS-444 [21] for 48 hours at 37°C. Cell survival was assayed using the MTT-based cell growth determination kit (Sigma-Aldrich) as previously described [3]. Relative cell survival was calculated as percentage relative to DMSO vehicle-treated controls. A dose-response curve was fitted to the data using the software KaleidaGraph 4.0 (Synergy), from which the half-maximal inhibitory concentration (IC_50_) was derived and represented as a mean of 4 independent experiments ± standard error of the mean (SEM).

### Annexin V staining and flow cytometry

HCT116 and RIE-1 cells treated with 10 μM MS-444 for 48 hours were subjected to Annexin V staining and flow cytometry per manufacturer's protocol (ThermoFisher-Molecular Probes). 1×10^6^ cells were resuspended in Annexin V binding buffer and incubated with 5 μL Annexin V-PE and 5 μL propidium iodide for 15 minutes in the dark. Cells were subjected to flow cytometry using the BD Accuri C6 flow cytometer. The collected data was subjected to compensation analysis by adjusting the gating and virtual voltage in the BD Accuri C6 software and graphically represented as means of 3 independent experiments. Cells treated with 5 μg/ml cycloheximide (Chx) (Sigma-Aldrich) and 10 ng/ml TNF-α (R&D Systems) for 8 hr were used as positive controls.

### Western blotting

Western blots were performed as described [12] using antibodies against HuR (clone 3A2, Santa Cruz Biotechnology) at a dilution of 1:20,000 for 1 hr at RT and COX-2 (160126, Cayman Chemical) at a dilution of 1:1000 for 16 hours at 4°C. Caspase 3 cleavage was detected using rabbit polyclonal anti-Caspase 3 (9662, Cell Signaling) at a dilution of 1:1000 for 16 hr at 4°C. Membranes were stripped and re-probed using β-actin antibody (Clone C4; MP Biomedicals). Cytoplasmic lysates were obtained using NE-PER cytoplasmic extraction reagent (Thermo Scientific) using α-tubulin (322500, 1:20,000 dilution, Invitrogen) and Lamin A/C (2032S, 1:2,000 dilution, Cell Signaling) as cytoplasmic and nuclear loading controls, respectively. Detection and quantitation of blots were carried out as described [10].

### RNA analysis

Total RNA was extracted using Trizol reagent (Invitrogen). cDNA synthesis was performed using 1 μg of total RNA in combination with oligo(dT) and Improm-II reverse transcriptase (Promega). qPCR analysis was performed as described [12] using the 7300 PCR Assay System with TaqMan probes for COX-2 (PTGS2) and GAPDH (Applied Biosystems). GAPDH was used as a control for normalization. Detection of apoptosis-associated mRNAs was accomplished using Apoptosis PCR Array PAHS-012Z (SA Biosciences/Qiagen) and qPCR was performed according to the manufacturer's protocol using SYBR green PCR master mix (Applied Biosystems). Fold change in mRNA expression levels was normalized to the cycle threshold (C_t_) using non-treated cells and analyzed by the ΔΔCt method.

### Ribonucleoprotein immunoprecipitations

Immunoprecipitation (IP) of ribonucleoprotein complexes (RNP-IP) was done as described [6] using HCA-7 cells grown on 100 mm dishes treated with either vehicle DMSO or 10 μM MS-444 for 6 hr. Prior to harvesting cells, 40 μl of Protein A/G beads (sc-2003, Santa Cruz Biotechnology) were coated with 20 μg of rabbit anti-HuR (3A2, Cat # sc-5261, Santa Cruz Biotechnology) or control IgG antibody overnight at 4°C. Beads were washed in NT2 buffer (50 mM Tris-HCl pH 7.4, 150 mM NaCl, 1 mM MgCl_2_, 0.05% NP-40) and incubated with equal amounts (250 μg) of cytoplasmic lysates obtained from cells lysed in 200 μl polysome lysis buffer (20 mM Tris-HCl pH 7.6, 5 mM MgCl_2_, 150 mM NaCl, 1 mM DTT, 0.5% NP-40) containing 100 U/ml RNase inhibitor (Ambion) and protease inhibitor cocktail (Sigma-Aldrich) and incubated overnight at 4°C with HuR- or control-IgG coated beads. Reactions were washed 5X with NT2 buffer and total RNA was isolated from immunoprecipitates using 1 mL TRIzol per IP reaction and then used for cDNA synthesis. Analysis of COX-2 (PTGS2) mRNA was done by qPCR as described above.

### DNA transfections

Luciferase reporter constructs containing the COX-2 3′UTR (Luc+COX-2 3′UTR) or control (LucΔ3′UTR) were transfected as previously described [31]. Cells were transfected for 24 hr, then treated with MS-444 for 8 hr before being lysed in reporter lysis buffer and assayed using the Luciferase Assay System (Promega, Madison, WI). Reporter gene activities were normalized to total protein; all results represent the average of triplicate experiments.

The MS2-based plasmids pcDNA-MS2-YFP and pMS2-COX-2 3′UTR used for fluorescent visualization of RNA, and the P-body marker mCherry-tagged Dcp1a (pmCherry-Dcp1a) have been previously described [31, 40]. The MS2 plasmids were transiently co-transfected with pmCherry-Dcp1a into HCT116 cells using Lipofectamine Plus for 24 hr and the cells were treated with 10 μM MS-444 for 8 hr. Cells were harvested in 2% paraformaldehyde and prepared for fluorescence microscopy.

### Immunofluorescence

Cells grown on coverslips were washed twice with PBS containing 10 mM glycine, fixed in 2% paraformaldehyde for 15 min at RT, and permeabilized with 0.02% Triton X-100 (Sigma-Aldrich) in PBS. Cells were blocked with 5% normal goat serum in PBS containing 1% IgG-free BSA (Jackson Immunoresearch) and incubated with primary antibody overnight at 4°C diluted in blocking solution. HuR was detected with anti-HuR monoclonal primary antibody (3A2, 1:200 dilution; Santa Cruz Biotechnology) for 1 hr at RT. Secondary antibody incubation was done for 1 hr at RT using FITC-conjugated anti-mouse IgG (1:100 dilution) or Alexa488-conjugated anti-mouse IgG (1:500 dilution; Invitrogen). Cells were counter-stained with DAPI to visualize nuclei. Fluorescence microscopy and image analysis was accomplished as described [31, 61]. Detection of P-bodies and assessment of P-body co-localization with MS2-YFP was accomplished as described [31].

### Xenograft tumor growth

Athymic nude (Nu/Nu) mice were purchased from Harlan Laboratories and maintained under sterile conditions in cage micro-isolators according to approved IACUC guidelines. HCT116 (2 × 10^6^ cells) and HCA-7 (2.5 × 10^6^) cells resuspended in PBS were injected into the dorsal subcutaneous tissue. Mice (n=5 per group) received intraperitoneal (IP) injections of MS-444 (25 mg/kg) dissolved in PBS/5% N-Methyl Pyrrolidine (NMP) (Sigma-Aldrich) or vehicle control every 48 hr. Tumor growth was assayed as described [3].

### Immunohistochemistry (IHC)

IHC was performed using formalin-fixed, paraffin-embedded (FFPE) tumors sectioned at 4 μm with monoclonal anti-HuR antibody (19F12; Molecular Probes) at 1:1250, polyclonal anti-cleaved caspase 3 (Asp175) antibody (#9661, Cell Signaling Technologies) at 1:1000, and polyclonal anti-COX-2 (160126; Cayman Chemical) at 1:400 dilutions. Staining and immunoreactivity scoring was performed as described previously [12, 65].

### Microvessel density analysis

Tumor microvessels were detected by IHC ofFFPE tumors using a polyclonal antibody to CD31 (ab28364; Abcam) at a dilution of 1:500 for 1 hr at RT in primary antibody diluent (Dako). After washing in Tris-buffered saline containing 0.05% Tween-20 (TBS-T), slides were incubated with biotinylated anti-rabbit labeled polymer (Dako). IHC was visualized using DAB peroxidase substrate kit (Dako) and counterstained with hematoxylin (Sigma-Aldrich). The area of tumor microvessels was quantitatively measured using the ImagePro Plus 4.5 software (Media Cybernetics) as previously described [66]. Tumor section images were imported into the ImagePro Plus software, where the CD31-positive staining was selected using the color selection function. Positive staining pixels were measured using the area/density (intensity) measurement function and represented as microvessel density.

### *Ex Vivo* imaging of COX-2

Athymic nude (Nu/Nu) mice bearing HCA-7 xenografts were established as described above. When palpable tumors (~100 mm^3^) were observed, mice were treated with MS-444 or vehicle control as indicated. COX-2 protein expression was visualized *ex vivo* by IP injection of 2 mg/kg of Fluorocoxib (Xenolght Rediject Fluorescent COX-2 Probe, Caliper Life Sciences) for 3 hr. Mice were euthanized and excised tumors were imaged in an IVIS platform (Caliper Life Sciences) with the following settings: 570 excitation, 620 emission, F-1 Stop, epifluorescence. Following visualization, the tumors were snap-frozen and processed for RNA and protein analysis.

### Statistical analysis

Statistical analyses were performed using GraphPad Prism (GraphPad Software). The data are expressed as the mean of three independent experiments ± S.E.M. Student's *t*-test was used to determine significant differences. P-values less than 0.05 were considered statistically significant.

## SUPPLEMENTARY MATERIALS FIGURES


