# Supplementary material for: Impact of HuR inhibition by the small molecule MS-444 on colorectal cancer cell tumorigenesis
Source: Oncotarget. 2016 Sep 22;7(45):74043–58. doi: 10.18632/oncotarget.12189 (PMC5342034; doi:10.18632/oncotarget.12189)
Supplement: Supplementary file 1 [file oncotarget-07-74043-s001.pdf]

Impact of HuR inhibition by the small molecule MS-444 on colorectal cancer cell tumorigenesis

SUPPLEMENTARY FIGURES

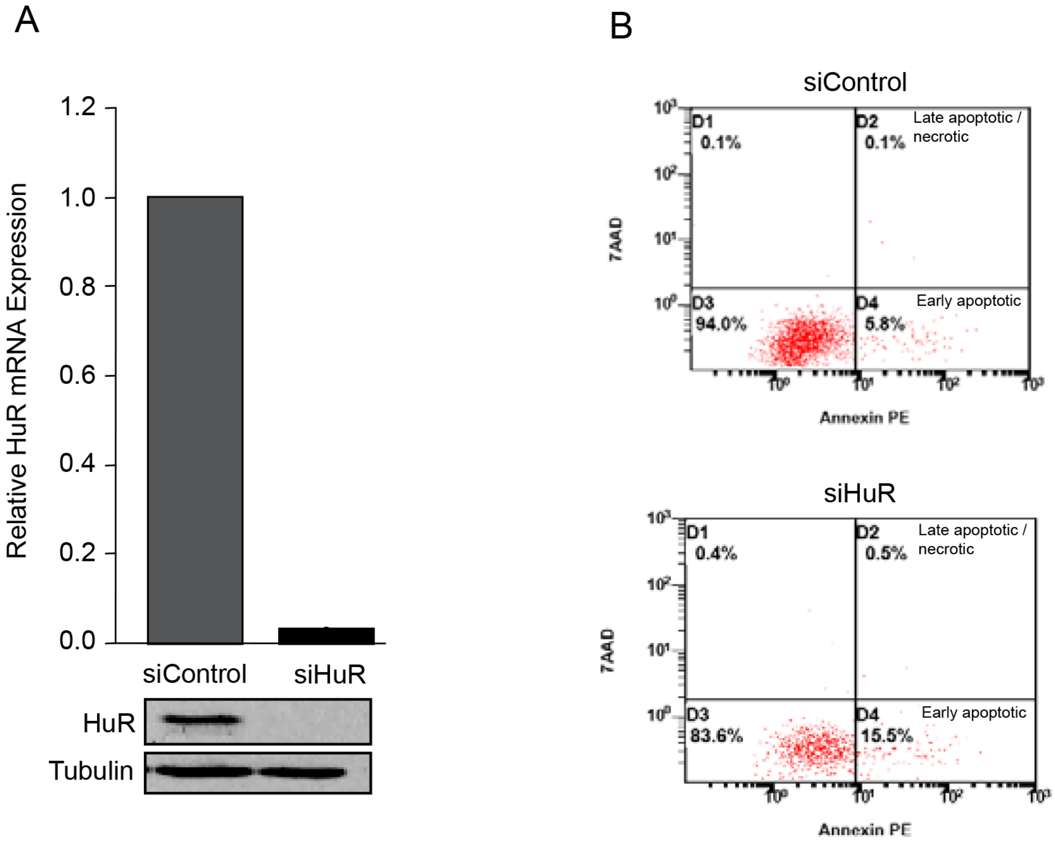

Supplementary Figure S1: siRNA knockdown of HuR expression promotes cellular apoptosis in colon cancer cells. A. HCT116 cells were transfected with a predesigned siRNA for HuR (siHuR) and negative control #1 siRNA (siControl; Ambion, Austin, TX) using siQUEST (Mirus, Madison, WI) for 48 hours according to the manufacturer's instructions. HuR mRNA levels were assayed by qPCR using GAPDH as a loading control. Results are represented as averages of triplicates ± SEM. HuR protein levels from HCT116 transfected were assayed by western blot. Tubulin was used as a loading control. **B.** HCT116 cells transfected with siControl or siHuR were stained with Annexin V-PE and 7-AAD and analyzed by flow cytometry in order to detect early apoptotic cells. Representative graph of experiment done in triplicate depicts the distribution of gated events.

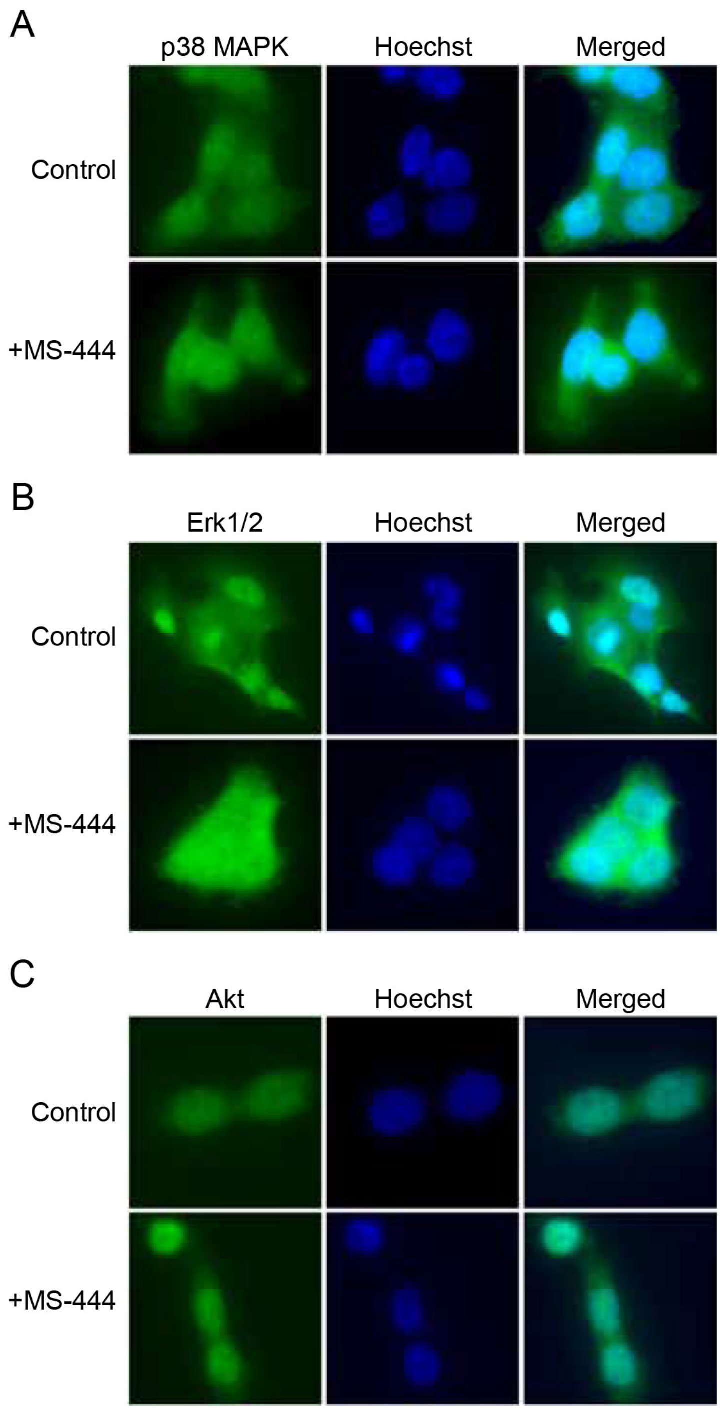

Supplementary Figure S2: MS-444 does not impact subcellular localization of p38 MAPK, Erk1/2, and Akt proteins. HCT116 cells were treated with DMSO control or 10 µM MS-444 for 6 hr and changes in subcellular localization of **A.** p38 MAPK, **B.** Erk1/2, and **(C)** Akt were assayed by immunofluorescence as described in Materials and Methods. Cells were counterstained with 1 µg/mL Hoechst 33342 (Sigma Aldrich) and were analyzed by fluorescence microscopy (40x).

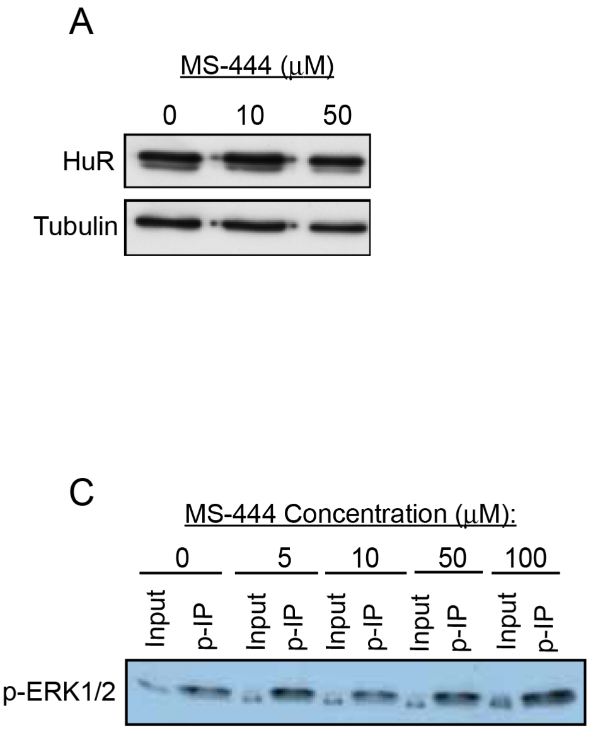

Supplementary Figure S3: MS-444 does not impact the expression of HuR or phosphoproteins in CRC cells. A. HCT116 cells were treated with 0, 10 µM, and 50 µM MS-444 for 24 hr and HuR protein levels were assayed by western blot. Tubulin was used as a loading control. **B.** HCT116 cells were treated with the indicated concentrations of MS-444 for 6 hr and western blot was performed to detect phosphorylated myosin light chain (MLC). Actin was used as a loading control. **C.** HCT116 cells were treated with the indicated concentrations of MS-444 for 6 hr and phosphoproteins (p-IP) were enriched from whole cell lysates using affinity columns as per manufacturer's protocol (Talon PMAC Phosphoprotein Enrichment Kit, Clontech, Mountain View, CA). Western blot was used to detect levels of p-ERK1/2.

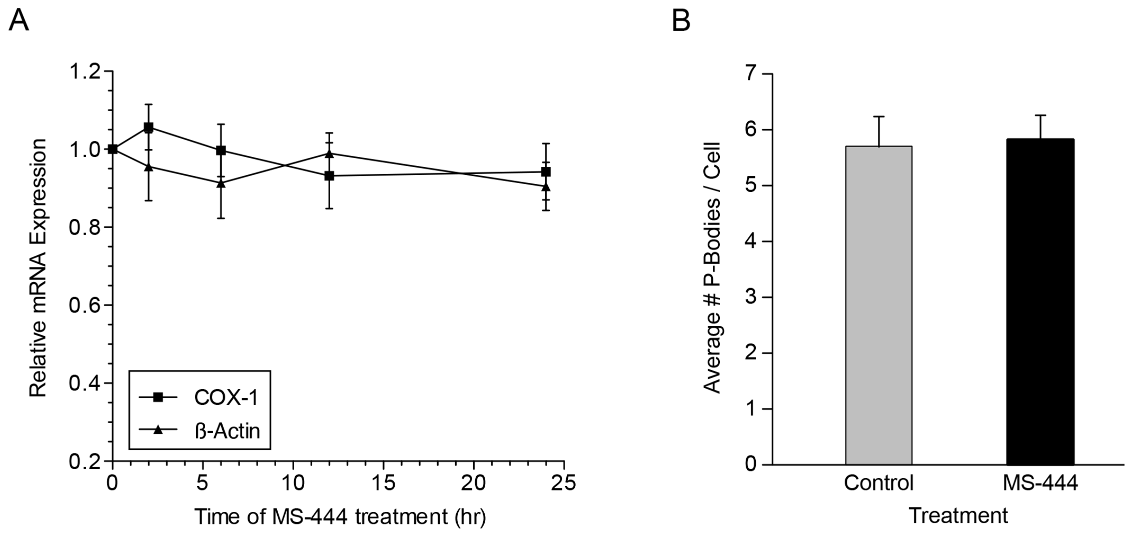

Supplementary Figure S4: MS-444 does not impact non-ARE mRNA turnover and P-body formation in CRC cells. A. HCA-7 cells were treated with 12 μM MS-444 or a vehicle control (DMSO) for the indicated times. COX-1 and β-actin mRNA levels were assayed by qPCR using GAPDH as a loading control and normalized to non-treated cells. Each value represents an average of triplicates ± SEM. **B.** HCT116 cells were transfected with mCherry-tagged Dcp1a to visualize P-bodies and treated with 10 μM MS-444 for 8 hr. P-bodies were assayed as described in Materials and Methods. Bar graph represents the average number of P-bodies per cell ± SEM (n = 10 cells per group).

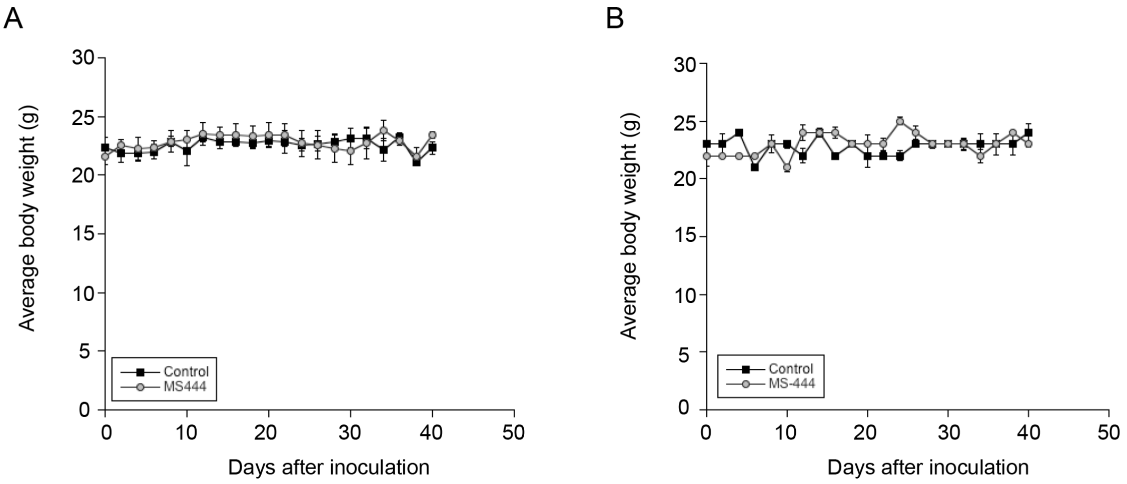

Supplementary Figure S5: *In vivo* effects of MS-444. Body weight measurements ± SEM for xenograft experiments of nude mice with **A.** HCT116 and **B.** HCA-7 tumors (n=5 mice per group). Mice received IP injections of MS-444 (25 mg/kg bw) or vehicle every 48 hr.
